# One-step preparation of antimicrobial silicone materials based on PDMS and salicylic acid: insights from spatially and temporally resolved techniques

**DOI:** 10.1038/s41522-021-00223-6

**Published:** 2021-06-21

**Authors:** Luca Barbieri, Ioritz Sorzabal Bellido, Alison J. Beckett, Ian A. Prior, Jo Fothergill, Yuri A. Diaz Fernandez, Rasmita Raval

**Affiliations:** 1 Open Innovation Hub for Antimicrobial Surfaces, Department of Chemistry, University of Liverpool & National Biofilm Innovation Centre, Liverpool, UK; 2 Institute of Infection and Global Health, University of Liverpool, Liverpool, UK; 3 Biomedical Electron Microscopy Unit, University of Liverpool, Liverpool, UK

**Keywords:** Biofilms, Antimicrobials, Applied microbiology

## Abstract

In this work, we introduce a one-step strategy that is suitable for continuous flow manufacturing of antimicrobial PDMS materials. The process is based on the intrinsic capacity of PDMS to react to certain organic solvents, which enables the incorporation of antimicrobial actives such as salicylic acid (SA), which has been approved for use in humans within pharmaceutical products. By combining different spectroscopic and imaging techniques, we show that the surface properties of PDMS remain unaffected while high doses of the SA are loaded inside the PDMS matrix. The SA can be subsequently released under physiological conditions, delivering a strong antibacterial activity. Furthermore, encapsulation of SA inside the PDMS matrix ensured a diffusion-controlled release that was tracked by spatially resolved Raman spectroscopy, Attenuated Total Reflectance IR (ATR-IR), and UV-Vis spectroscopy. The biological activity of the new material was evaluated directly at the surface and in the planktonic state against model pathogenic bacteria, combining confocal laser scanning microscopy, electron microscopy, and cell viability assays. The results showed complete planktonic inhibition for clinically relevant strains of *Staphylococcus aureus* and *Escherichia coli*, and a reduction of up to 4 orders of magnitude for viable sessile cells, demonstrating the efficacy of these surfaces in preventing the initial stages of biofilm formation. Our approach adds a new option to existing strategies for the antimicrobial functionalisation of a wide range of products such as catheters, wound dressings and in-dwelling medical devices based on PDMS.

## Introduction

Polydimethylsiloxane (PDMS) is a silicon-based polymer commonly used in biomedical and industrial applications^1–3^, including the fabrication and prototyping of medical devices^4–7^, and the formulation of pharmaceutical, cosmetic and food products (regulated food additive E900) (European Union Regulation (EC) No 1333/2008 of the European Parliament and of the Council of 16 December 2008 on food additives in force 02/07/2020, (2020) Document 02008R1333-20200702, data.europa.eu/eli/reg/2008/1333/2020-07-02). The surface of PDMS is highly hydrophobic and almost unreactive due to the presence of surface-oriented methyl groups from the dimethylsiloxane moiety^8,9^. Therefore, covalent functionalisation of PDMS devices with antimicrobial components can be challenging, often requiring complex and invasive approaches that cannot be easily translated from lab-scale into industrial production lines within strict regulatory environments. The use of antimicrobial coatings on PDMS devices has been explored before^10–12^; however in-depth characterisation of surface and sub-surface distributions of active molecules is scarce, limiting the development of knowledge-based design strategies for industrial scale processes.

The market for PDMS-based devices is continuously growing, with academic and industrial communities developing materials for cutting-edge applications, including anti-biofouling coatings^3^, microfluidic devices^2^ and soft-electronics^13^. However, translation into applications is limited by the lack of simple manufacturing methods that can be implemented at industrial scale with minimal impact on existing production pipelines. The approach proposed in this paper overcomes some of these translational obstacles by combining chemical components already approved for pharmaceutical and cosmetic applications within a sequential manufacturing process that relies exclusively on scalable technologies. Furthermore, our detailed characterisation of the chemical properties and the performance of the new material demonstrates reliability and robustness of the manufacturing protocol. We envision that this alternative approach will allow the functionalisation of PDMS-based medical devices with different active components, opening opportunities for novel technological applications.

## Results and discussion

### Fabrication and characterisation of PDMS-SA materials

**Fig. 1:**
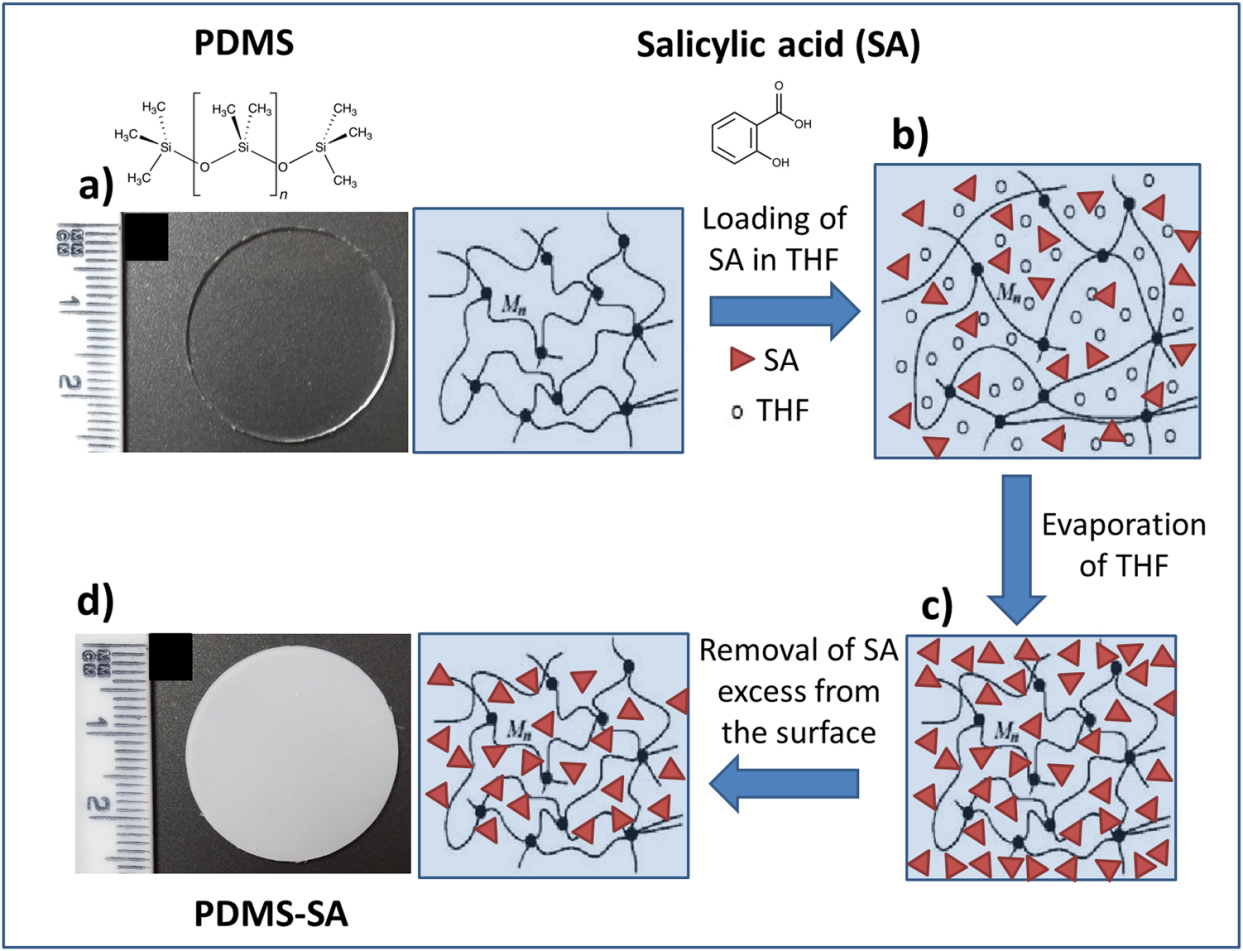
Schematic representation of the loading process of salicylic acid into the polymeric matrix of PDMS. **a** Pristine PDMS; **b** swelled PDMS in the presence of a concentrated solution of SA in THF; **c** fast evaporation of THF leaves SA trapped within PDMS and at the surface; **d** removal of excess of SA from the surface leads to PDMS-SA samples. Inserted optical images show pristine PDMS and functionalised PDMS-SA samples.

PDMS is widely used in several applications because it is chemically inert, but this characteristic also imposes big challenges for the chemical functionalisation of PDMS devices. Often, the incorporation of chemical moieties into the polymeric backbone of cured PDMS can be only achieved under strong oxidising environments or using reactive plasma reactions^14^. Here we utilise an alternative route, exploiting the ability of PDMS to swell in the presence of specific organic solvents^15^ to incorporate a known antimicrobial compound, salicylic acid (SA), into PDMS materials without altering the chemical properties of the polymer. Diffusion of SA across PDMS membranes has been observed in the presence of simple organic solvents^16^ suggesting that transport of SA into the bulk of PDMS should be feasible. Figure 1 illustrates the main aspects of our approach.

We specifically selected tetrahydrofuran (THF) as the carrier solvent, in view of the high ratio of swelling of PDMS under this solvent^15^ and the elevated solubility of SA, allowing high doses of the biocide to be handled. Furthermore, its high volatility allows fast evaporation of THF at room temperature, ensuring its complete removal and a relatively low toxicity of the functional PDMS material, desirable for healthcare applications.

The loading process, described in Fig. 1, encompasses an initial stage where pristine PDMS (Fig. 1a) is set in equilibrium with a nearly saturated solution of SA in THF. This initial step allows a considerable swelling of the PDMS matrix induced by THF, carrying high concentrations of SA into the swelled polymeric matrix by osmosis (Fig. 1b). After swelling, and upon fast evaporation of THF, the non-volatile molecules of SA are left behind within the interstitial space of the swelled polymeric backbone of PDMS and at the surface of the sample (Fig. 1c). Removal of the surface excess of biocide was achieved by gentle mechanical abrasion and subsequent rinsing with a saturated aqueous solution of SA under sonication, leading to PDMS-SA samples. This simple process enabled the fabrication of PDMS-SA samples containing high concentrations of SA trapped within the polymer with the loading process enabling incorporation of 4.5% (±0.1%) in weight of SA into PDMS samples, as determined directly by gravimetric analysis. The presence of SA was also visible by a change in colour of the samples (see optical images in Fig. 1). The procedure is based on a series of discrete operations that can be easily adapted and scaled up into industrial production lines, allowing direct translation of the technology for the functionalisation of pre-manufactured PDMS devices.

**Fig. 2:**
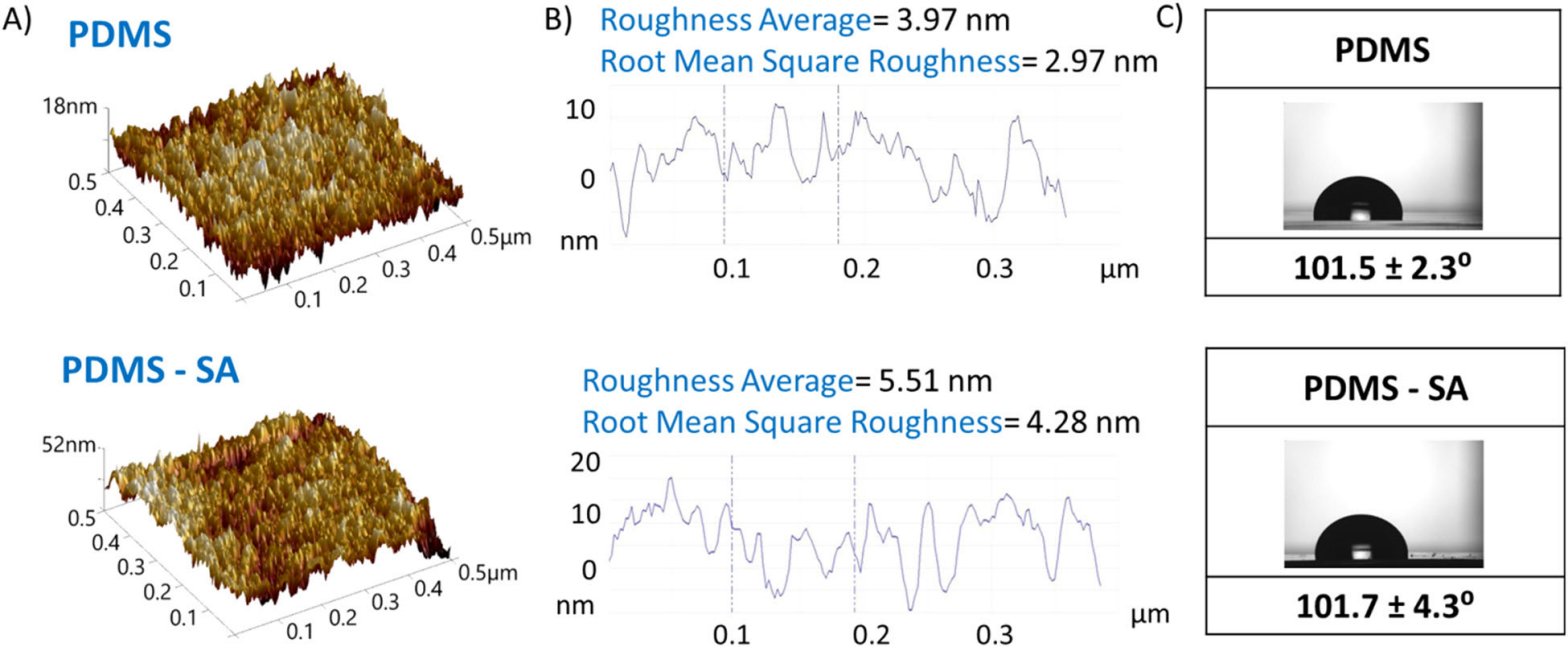
Surface properties of PDMS and PDMS-SA samples. **A** AFM images showing the topography of the surfaces. **B** Surface profile and roughness of the surfaces obtained from AFM data. **C** Water contact angle at the surfaces.

Additionally, the SA loading process did not affect key surface properties of PDMS. The surface roughness of PDMS samples, as determined by AFM, showed only small variations after incorporation of SA (Fig. 2a, b). Similarly, the wettability of the PDMS surface was not significantly altered by the incorporation of SA, displaying contact angles typical of hydrophobic surfaces (e.g. >100°), Fig. 2c. The PDMS-SA samples also preserved a good elasticity and showed mechanical properties that resembled those of pristine PDMS.

**Fig. 3:**
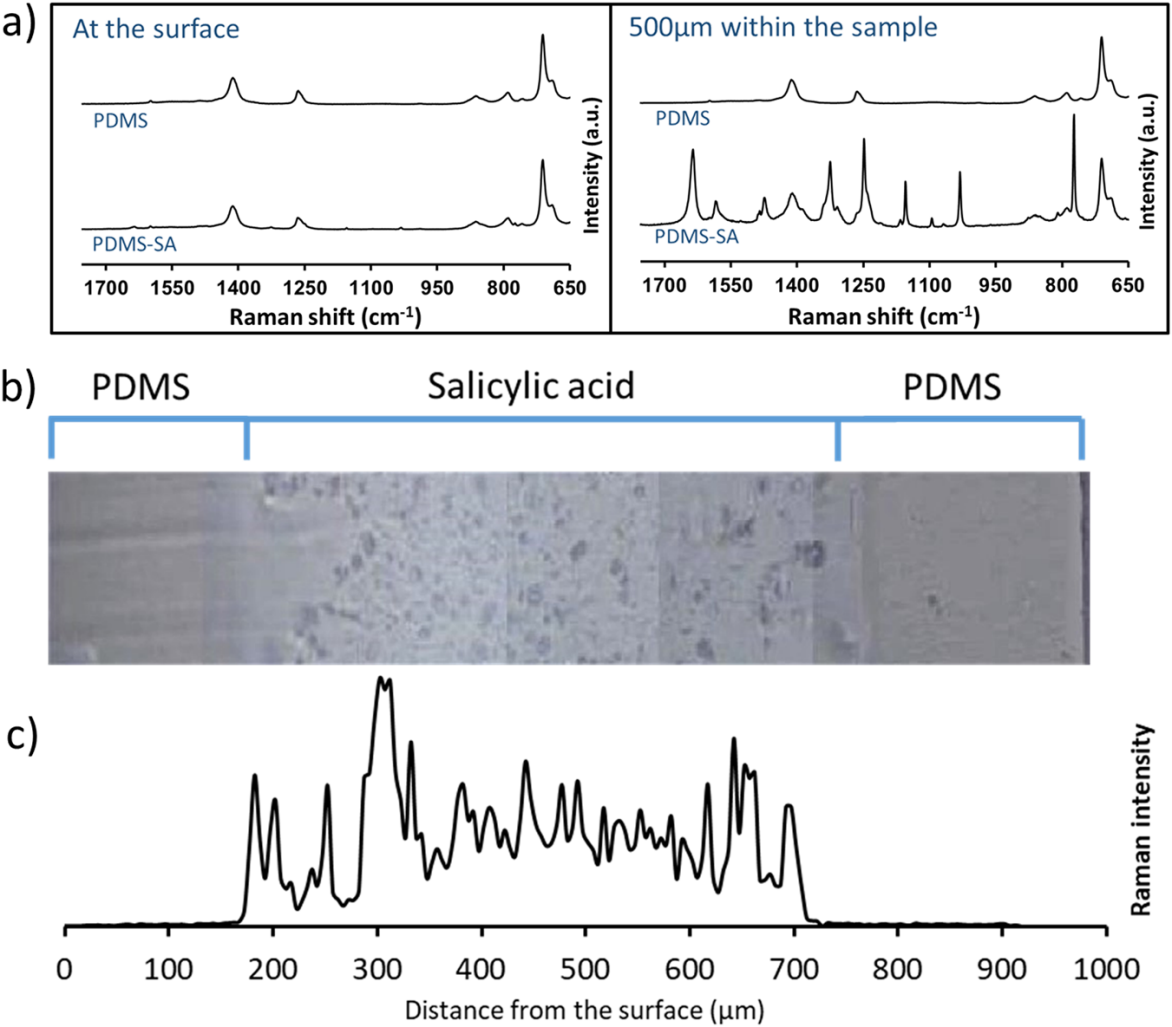
Spatially resolved Raman spectroscopy of PDMS and PDMS-SA. **a** comparison of Raman spectra at the surface and at 500 µm within the sample; **b** Optical image of the cross section of a representative PDMS-SA sample; **c** Raman intensity of SA peak at 1032 cm^−1^ along the cross-section of PDMS-SA.

The location of SA within PDMS-SA samples was directly probed by spatially resolved Raman spectroscopy^16^. Figure 3a shows representative spectra of PDMS and PDMS-SA samples taken at the surface and at a depth of 500 µm within the samples. The characteristic Raman peaks for SA were clearly observed in spectra taken 500 µm within the PDMS-SA samples, but were barely seen at the sample surface (Fig. 3a), suggesting that the biocide is located predominantly inside the polymeric matrix, away from the surface. In order to confirm these findings we conducted detailed Raman characterisation of cross-sections of PDMS-SA samples. For this analysis we selected the Raman peak of SA at 1032 cm^−1^ to avoid interference with the main peaks of PDMS, observed above 1200 cm^−1^ and below 900 cm^−1^. The analysis of the distribution of SA at the cross-section of PDMS-SA samples (Fig. 3c), confirmed that the biocide is mainly present within a confined region of the bulk sample with the onset starting at a depth of ~150 µm away from the sample surface.

### Probing the release of SA from PDMS-SA samples

**Fig. 4:**
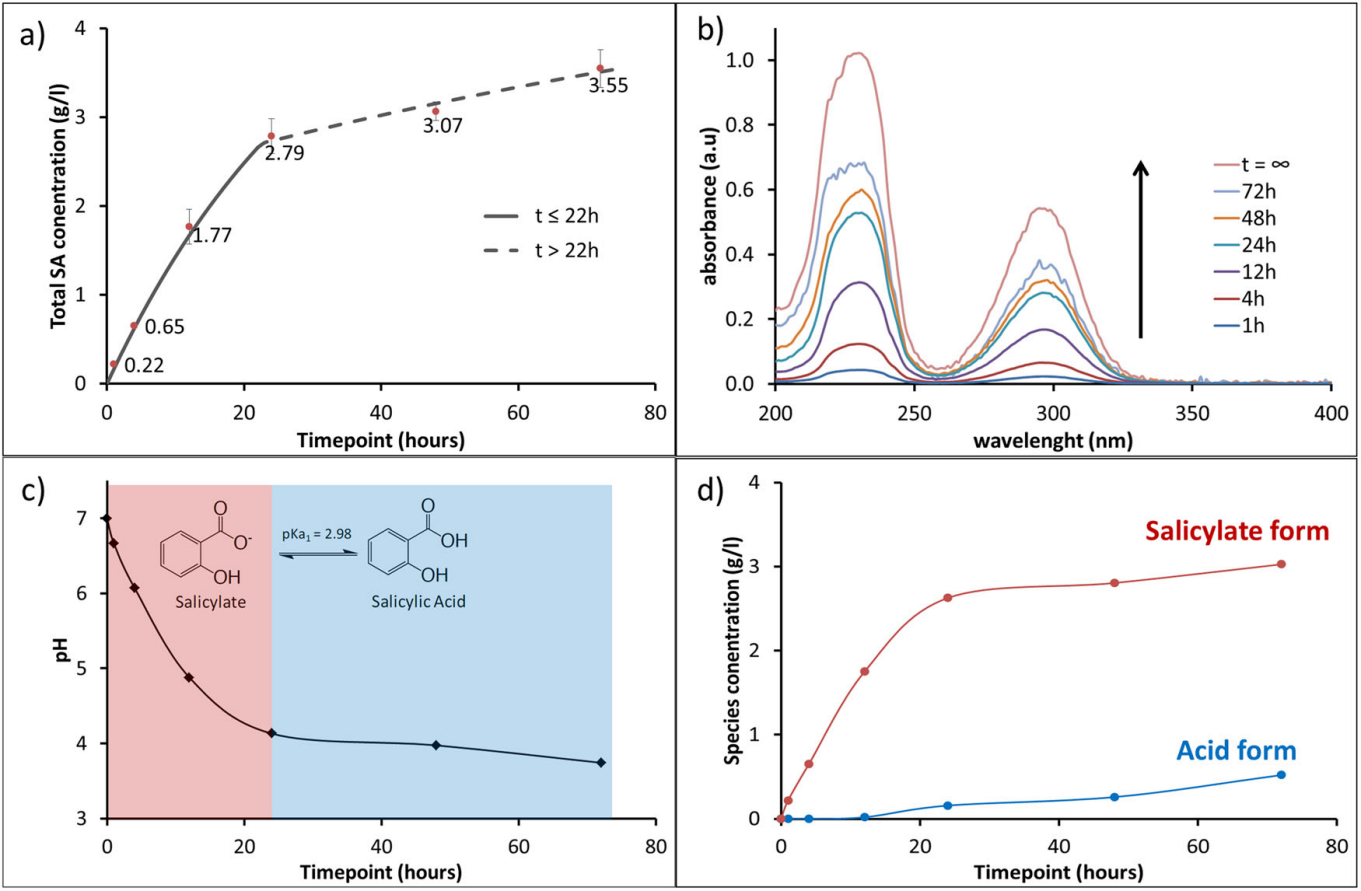
Kinetics of SA release into the liquid media probed by UV-Vis spectroscopy. **a** Total release of SA from PDMS-SA samples showing two kinetic regimes; **b** Representative spectra of the released SA solutions at different time points, with vertical arrow indicating the increased concentration as a function of time and *t*_∞_ showing the spectrum of a saturated SA solution; **c** pH of the release solution as a function of time; **d** Concentration of SA species in the release solution, i.e. protonated acid form and deprotonated salicylate as a function of time.

The results presented in the previous section demonstrated that in PDMS-SA samples, the biocide is mainly located within the polymeric matrix, away from the surface. In order to probe if the encapsulated biocide is available for release, we investigated the concentration of released SA within liquid media in contact with PDMS-SA at different time points. For these experiments we used a model buffer based on sodium citrate and citric acid that accurately reproduces the pH response of typical growth media for bacterial culture while being optically transparent in the UV-Vis spectral region (see Supplementary Figs. S5.1 and S5.2). This model buffer allowed direct quantification of released SA in the liquid media, after exposure to PDMS-SA samples, using UV-Vis spectroscopy. Our results showed that, despite the biocide’s location away from the surface, SA can be released from PDMS-SA samples, reaching concentrations over 3 g/L after 72 h (Fig. 4a, b).

Interestingly, the released profile of SA showed two kinetic regimes, below and above the critical time of ≈22 h, that can be associated with two different release constants within the first-order expression:

1 $$\frac{{{\mathrm{d}}C_{{\mathrm{SA}}}}}{{{\mathrm{d}}t}} = k_{\mathrm{i}}\left( {S_{{\mathrm{SA}}} - C_{{\mathrm{SA}}}} \right)$$

here *C*_SA_ is the time-dependent concentration of biocide in the liquid media, *S*_SA_ is the solubility of SA, and *k*_i_ is the apparent kinetic constant for the release process at the specific kinetic regime, i.e. *k*_1_ for *t* < 22 h and *k*_2_ for *t* > 22 h, but both following Eq.1 in different time intervals. This expression resembles a saturation-limited solubilisation process, in which the rate of release decreases as the concentration of the solute in the media approaches the solubility and becomes effectively zero when equilibrium is reached (i.e. the equilibrium condition is *C*_SA_ = *S*_SA_ at *t* = ∞). We determined the solubility of SA in the model buffer in independent experiments, obtaining *S*_SA_ = 5.43 g/L. The apparent release constants, *k*_1_ = 0.030 s^−1^ and *k*_2_ = 0.007 s^−1^ for *t* < 22 h and *t* > 22 h respectively, were obtained by linearisation of the analytical solution of Eq.1 and using this solubility value for SA (details discussed in Supplementary Information, section SI6).

The two release regimes observed can be rationalised by considering the pH change in the liquid media as a result of SA release (Fig. 4c). Initially, the buffer solution is pH = 7 and, as SA is released from PDMS-SA, the pH progressively decreases, reaching the intrinsic buffering-region for SA at pH ≈ 4 (i.e. pH ≈pKa ± 1, pKa = 2.98^17^). Our data showed that this transition into the buffering-region of SA occurs around the critical time of 22 h and critical pH ≈ 4, coinciding with the change in kinetic regime observed for SA release. Taking this evidence into account, we can explain the two release regimes as follows: at pH values well above the SA buffering-region (*t* < 22 h, pH > 4), SA molecules are predominately present in the deprotonated salicylate form, displaying higher solubility in water and fast release rates (*k*_1_ = 0.030 s^−1^). When the pH buffering-region of SA is reached (*t* > 22 h, pH < 4), a fraction of SA molecules is protonated (Fig. 4d), displaying low solubility in water and, consequently, slower release rates (*k*_2_ = 0.007 s^−1^). Irrespective of the complexity of this release profile, our results demonstrated that during the initial phase of fast release, SA concentrations reached values above 2 g/L within the first 24 h and thereafter showed a steady maintenance release for over 72 h without reaching saturation or depletion of the SA reservoir within the sample. An important point to note is that this release profile is highly advantageous, providing an initial peak of fast biocide release rate, followed by long-term sustained concentration.

### Mapping the distribution of sub-surface SA as a function of release time

**Fig. 5:**
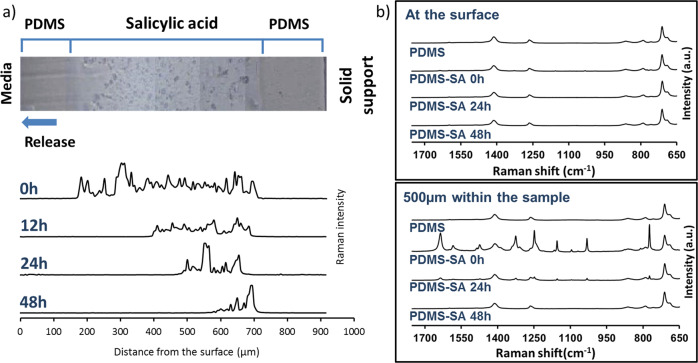
Raman characterisation of PDMS-SA surface and bulk after SA release at different time points. **a** SA distribution along the cross section of PDMS-SA as a function of release time probed by confocal Raman microscopy. **b** Comparison of time evolution for the Raman spectra at the surface and at 500 µm within the sample.

To unravel the details of the release of SA from PDMS-SA, we subsequently investigated the cross-sections of the functionalised samples at different time points using Raman confocal microscopy. By mapping the intensity of the characteristic peak of SA at 1032 cm^−1^ we were able to evaluate the time evolution of SA distribution within the cross-section of PDMS-SA samples. The results (Fig. 5a) confirmed that SA was initially located inside the bulk material of the PDMS-SA samples, and as the release process progressed with time, the SA signal progressively retreated inwards, resembling an advancing solvent front. This evidence points to the penetration of the aqueous liquid media into the sample, enabling the release of the biocide.

**Fig. 6:**
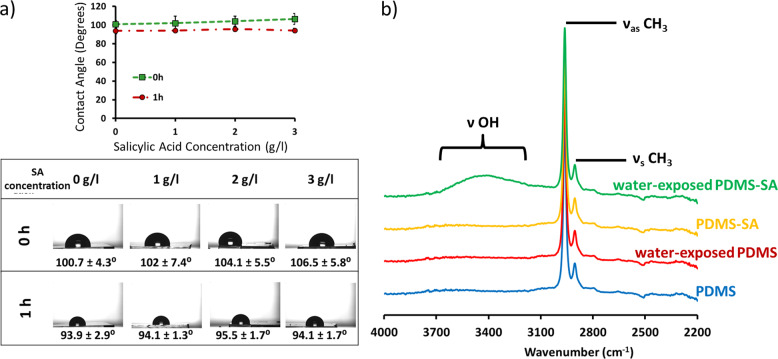
Effect of SA on surface properties of PDMS and PDMS-SA samples. **a** Static contact angle for the model buffer containing different concentrations of Salicylic acid (SA) over PDMS at contact times of 0 h and 1 h, showing that the presence of SA in the media does not affect the wettability of PDMS surface; **b** ATR-FTIR spectra of PDMS and PDMS-SA samples before and after 24 h of contact with the aqueous model buffer, simulating a typical release experiment.

The interpretation of our Raman cross-section data was further confirmed by attenuated total reflectance–Fourier transform infrared (ATR-FTIR) measurements revealing the presence of the νOH vibrational peaks of water^18^ in the PDMS-SA samples exposed to the aqueous media (Fig. 6b). Water was not present on pristine PDMS exposed to the aqueous media, confirming that pristine PDMS is inherently impermeable to water. Therefore, it appears that the presence of SA within the polymeric matrix of PDMS may change the local permeability of the polymer, allowing water to penetrate into the polymer bulk. We also noted that these local changes had little effect on the average surface properties of PDMS, reflected in the nearly constant value of the contact angle of PDMS-SA samples before and after exposure to water (see Supplementary Fig. S1.1 and S1.2 for additional contact angle data). Similarly, we observed no effect of the dissolved SA in the surface wettability (Fig. 6a), suggesting that the unexpected permeability of PDMS-SA cannot be attributed to the presence of SA in the liquid media.

These results allow us to propose the following mechanism for the release of the biocide: the presence of SA within PDMS increases the penetration capacity of water into the PDMS matrix, allowing dissolution of solid SA trapped inside PDMS-SA and, subsequently, its diffusion and release from the surface to the aqueous media.

### Antimicrobial activity in the planktonic state

**Fig. 7:**
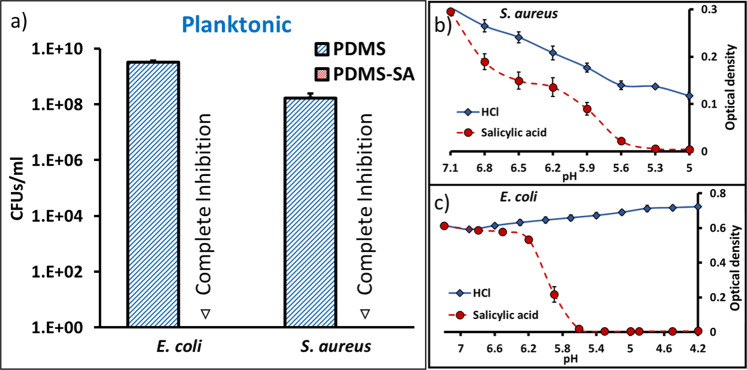
Inhibition of planktonic *E. coli* and *S. aureus* after 24 h of incubation. **a** Antimicrobial activity of PDMS-SA compared to PDMS control samples, inverted triangles indicate complete inhibition at inoculum 10^4^ CFU/ml (additional data for higher inoculum is presented in Supplementary Information); **b**, **c** Deconvolution of pH effects on bacterial growth for **b** *E. coli* and **c** *S. aureus* incubated in growth medium with increasing concentrations of hydrochloric acid (HCl) or SA, showing that the minimum inhibitory concentration for SA is reached at pH values where bacterial growth is still possible.

After demonstrating the release of sub-surface SA within PDMS-SA materials, we subsequently investigated the antimicrobial activity of these materials against two model microorganisms, *Escherichia coli* and *Staphylococcus aureus* (i.e. *E. coli* strain J96 and *S. aureus* strain SH1000), which are relevant clinical pathogens^19–21^. For this purpose we determined the number of colony forming units (CFUs) in the planktonic media after 24 h of contact with the PDMS-SA samples. Pristine un-functionalised PDMS samples were used as controls for these experiments. The results showed significant inhibition of planktonic growth for both *E. coli* and *S. aureus* (Fig. 7a and Supplementary Fig. S8.2).

This significant antibacterial performance can be explained by the fact that the biocide released from PDMS-SA samples reaches concentrations above the minimum biocidal concentrations (MBCs) for both *E. coli* and *S. aureus* after 24 h of incubation (MBC 2.00 g/l and 1.75 g/l for *E. coli* and *S. aureus*, respectively). We note that the effect of SA at these concentrations cannot be solely attributed to the change of pH in the growth medium, but to an intrinsic antimicrobial effect of the biocide. This was assessed experimentally by adding only HCl to the growth medium to reach pH values equivalent to the MBC of SA, which showed that bacterial growth was not inhibited by the change in pH (Fig. 7b, c and Supplementary Fig. SI4.1 for additional bacterial growth curves).

### Antimicrobial activity at the surface

Sessile bacteria are generally less susceptible to environmental stress and antimicrobial compounds compared to planktonic microorganisms. The enhanced resistance of sessile bacteria to biocides can lead to persistent infections associated with surface borne biofilms^22^. For this reason, we also investigated the antimicrobial effect of PDMS-SA materials on sessile bacteria, directly attached to the surface, combining complementary microscopy techniques and quantitative analysis of sessile CFU.

**Fig. 8:**
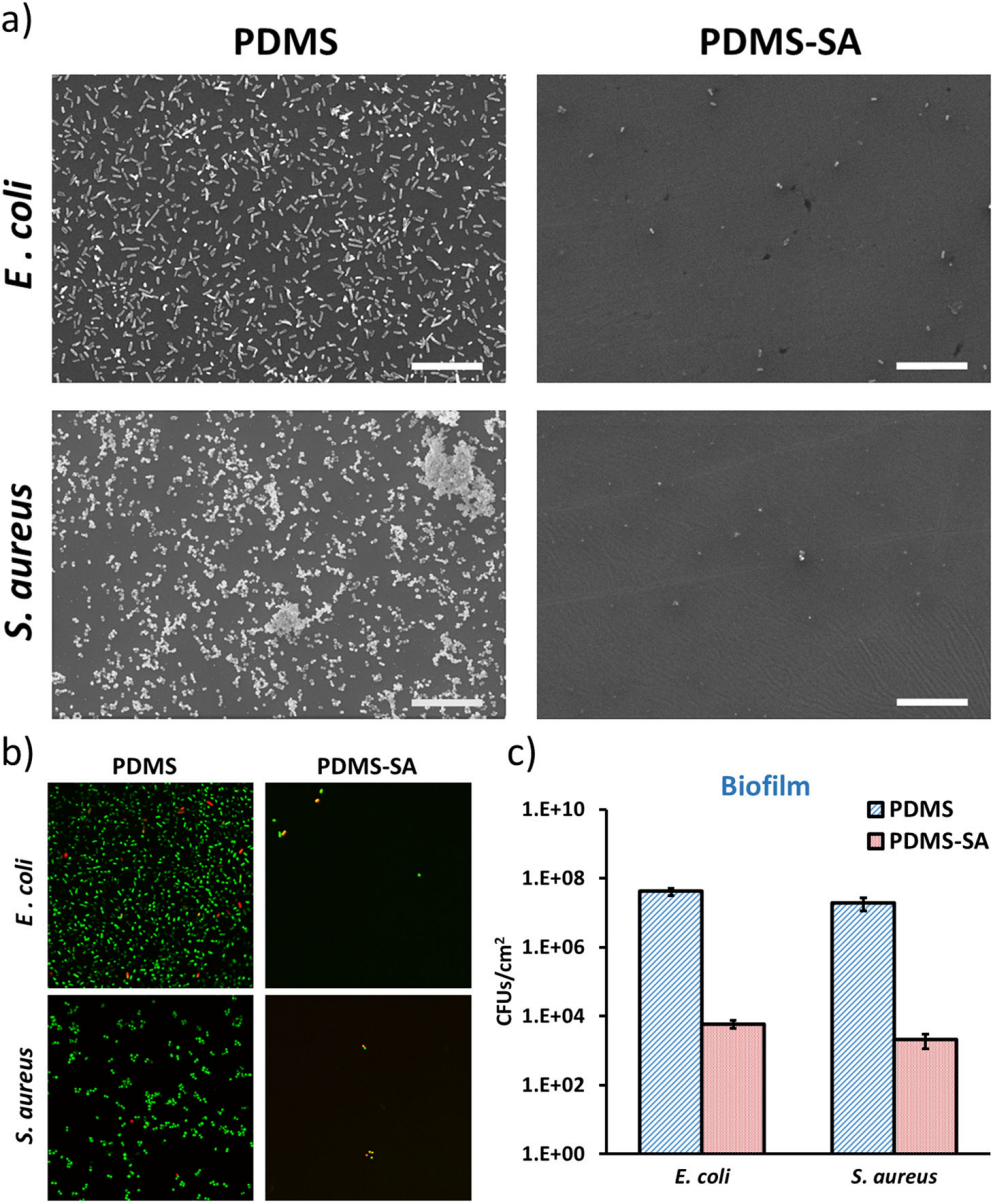
Antimicrobial activity of PDMS-SA samples against sessile *E. coli* and *S. aureus* cells compared to PDMS control samples. **a** Representative scanning electron microscopy images of *E. coli* and *S. aureus* at the surface of PDMS and PDMS-SA samples after 24 h of incubation (scale bar 20 µm); **b** Representative confocal fluorescence images of bacteria at the surface of PDMS and PDMS-SA samples after 24 h, as stained by Live/Dead fluorescent labelling, showing live cells in green and dead cells in red/yellow (image size 71 µm × 71 µm); **c** Inhibition of sessile *E. coli* and *S. aureus* cells after 24 h of incubation (inoculum 10^4^ CFU/ml) on PDMS-SA.

Surface coverage was initially evaluated by scanning electron microscopy (SEM). We have previously optimised specific methods for SEM sample preparation, allowing us to preserve delicate bio-interfaces and enabling direct imaging of biofilms and single cells attached to functionalised surfaces^23,24^. Here, we applied those methods to PDMS and PDMS-SA samples incubated for 24 h with the model bacteria *E. coli* and *S. aureus*. The SEM images showed that both strains were able to colonise the control PDMS samples, leading to widespread coverage of the surface, characterised by some areas of singe-cell attachments and other zones of multi-layered biofilms (Fig. 8a). Conversely, PDMS-SA surfaces showed a considerably reduced density of cells, with only residual coverage by isolated single-cells, spread over the sample surface.

Similar results were observed by confocal fluorescence microscopy, using Live/Dead staining with Syto 9 and propidium iodide. Syto 9 (green channel in Fig. 8b) is able to penetrate the membrane of all the cells at the surface, while propidium iodide (red channel in Fig. 8b) exclusively stains cells with compromised membranes, i.e. the dead cells. With this technique, we were able to probe the viability of the remaining cells attached to the surface. Our data on Fig. 8b, demonstrated that within the low residual coverages observed on PDMS-SA samples, many individual cells were already dead (i.e. cells stained red/yellow). The total number of sessile cells on the active surfaces was too small for an accurate quantification of this effect by direct microscopy at the surface. For quantitative determination we relied on the analysis of sessile CFU.

The surface density of sessile CFUs was determined for both control PDMS and PDMS-SA samples incubated for 24 h in bacterial cultures, by accurate dispersion of the biofilm and subsequent enumeration. The coverage on control PDMS surfaces was between 10^6^ and 10^8^ CFU/cm^2^ for both bacterial strains investigated here and for the two different inoculum concentrations used (Fig. 8c and Supplementary Fig. S8.1). This surface coverage dropped by a log_10_ reduction factor ≈4 for both bacteria on PDMS-SA samples with inoculum concentrations 10^4^ CFU/ml. We observed relatively smaller yet significant effects for higher inoculum concentrations of 10^5^ CFU/ml (i.e. log_10_ reduction factors between 2 and 3). These results confirm that the antimicrobial activity of these functionalised materials can be effectively delivered at the surface.

## Conclusions

We have shown that highly effective antimicrobial PDMS materials can be generated via the incorporation of salicylic acid (SA). The significant reduction of up to 4 orders of magnitude on the viability of bacteria at the surface, combined with complete inhibition on planktonic cells demonstrates that these materials can be effectively used to prevent the initial stages of biofilm formation on PDMS devices and reduce the risk of chronic infections. The simplicity of the fabrication method proposed here, based exclusively on unit chemical operations, combined with the low regulatory barriers for all materials and chemicals used, pave the way for a direct translation of the technology into industrial-scale applications. Additionally, our spatially resolved characterisation techniques have enabled us to track the release of the biocide from the bulk of the material into the surrounding media. The release profile of our materials combines an initial peak of fast biocide release rate, followed by long-term sustained concentration, providing advantages compared with existing strategies for prevention and eradication of infections in health-care settings, with applications across a wide range of products such as catheters, wound dressings and in-dwelling medical devices.

## Methods

### Fabrication of PDMS-Salicylic acid samples

PDMS (Sylgard 184 silicone elastomer kit, Dow Corning) discs were fabricated mixing 10 parts of base and 1 part of curing agent, then, 1.1 g of the resulting mixture were poured to individual wells of a six-well plate and cured overnight at 50 °C. The discs were then removed from the six-well plate and left overnight at 4 °C in a 500 mg/ml solution of Salicylic Acid (SA) (Alfa Aesar) in Tetrahydrofuran (THF) (Sigma-Aldrich). The excess of SA was mechanically removed from the surface and the samples were then sonicated in a nearly saturated solution of SA in DI water for 30 min at room temperature. This washing procedure was repeated three times changing the SA saturated solution. The materials were then removed from the solution, allowed to dry and cut in 2.5 cm diameter discs. In all tests reported in this work, the PDMS-SA samples were used the day after the cleaning procedure. The amount of SA loaded into the samples was determined by gravimetric analysis in triplicate, leading a loading capacity of 4.5% ± 0.1% (w/w) of loaded SA into PDMS.

### Raman microscopy analysis

Surface Raman spectra were recorded using a Renishaw inVia Upright Confocal Raman microscope using an excitation wavelength of 532 nm and a ×20 Leica objective lens. Laser power ranged from 50 to 100% and collection time varied from 5 s to 30 s, depending on the different types of sample, to achieve good signal to noise ratios while avoiding saturation. The Raman spectra were processed and normalised for comparison using Wire 4.4 analysis software (Renishaw). Samples were analysed from the top of the sample, focusing first at the surface and moving the focal plane 500 µm within the bulk of the material. Additionally, lateral cross-sections were investigated by cutting the samples and acquiring one spectrum every 20 µm within an imaginary straight-line crossing from the top surface to the bottom of the samples. The distribution of SA at the cross-section was reconstructed from these lines of spectra by integrating a characteristic peak of SA (i.e. from 1020 cm^−1^ to 1045 cm^−1^) normalised with respect to the reference PDMS peak (i.e. from 690 cm^−1^ to 720 cm^−1^).

### Attenuated total reflectance – Fourier transform infrared

The presence of water in PDMS-SA and pristine PDMS samples after contact with the aqueous model buffer was investigated by ATR-FTIR spectroscopy. Two sets of samples were analysed, namely dry samples (with no contact with liquid media after the cleaning process) and “water-exposed” samples, kept for 24 h in 4 ml of release media at 37 °C. ATR-FTIR spectra were acquired using a Bruker Alpha FT-IR with Platinum ATR module with a resolution of 4 cm^−1^. Each spectrum was the average of 24 scans. Sample spectra were collected within 400 cm^−1^ and 4000 cm^−1^ range and analysed using OPUS Spectroscopy Software (Bruker). SA was not detected by ATR-FTIR on any of the samples due to the limited depth penetration of the ATR configuration, allowing to probe only the sample interface, far from the specific location of SA within PDMS-SA bulk, i.e. ≈150 µm away from the surface.

### Contact angle measurements

Surface wettability was assessed by static contact angle measurements, using a goniometer CAM 100, KSV Instruments with an accuracy of 0.1°. We performed at least three measurements in different areas within each PDMS and PDMS-SA surface, obtaining reproducible results. The contact angle of a compressed-pellet of SA was also measured. We also performed contact angle experiments with different concentrations of SA and specific treatments of the samples to probe any effect on surface wettability. In all these contact angle experiments we used the aqueous model buffer based on citrate and citric acid as the probing liquid.

### Atomic force microscopy analysis

Atomic force microscopy (AFM) was used to probe the topography of PDMS and PDMS-SA surfaces using a Bruker Multimode 8 AFM fitted with a NanoScope controller operating in a ScanAsyst mode and equipped with a silicon tip and a cantilever operating at a scan rate of 0.977 Hz. AFM images were taken with a 512 × 512 pixels resolution and were analysed using Nanoscope analysis software. The average roughness (Ra) and root-mean-squared roughness (Rq) were determined from images at 0.5 mm×0.5 mm scan size.

### Release of SA from PDMS-SA samples

The concentration of released SA was evaluated using UV-VIS Spectrophotometry in conditions similar to those of the bacterial viability experiments. The bacterial growing media (Luria-Bertani nutrient broth) was substituted in this case by a model buffer transparent in the UV-Vis region of the spectra. The model buffer was prepared by dissolving 4.6 mM citric acid, 6.9 mM sodium citrate tribasic dehydrate in DI water. The final pH of this model buffer was adjusted to pH 7 using a 1-M sodium hydroxide solution (Sigma Aldrich). For the release experiments, each PDMS-SA sample was incubated at 37 °C in individual wells within six-well plates, containing 4 ml of model buffer per well. The SA concentration was assessed at different time points. The released concentration was determined using the extinction coefficient for SA *ε* = 24.78 L/g at 299 nm. We also determined the solubility of SA in the model buffer by means of UV-Vis spectroscopy on a saturated SA solution.

### Antimicrobial testing

For both planktonic and surface viability test, bacteria (*S. aureus* SH1000 strain and *E. coli* J96 strain) were transferred from frozen stocks to sterile nutrient agar plates and incubated overnight at 37 °C. Subsequently, three individual colonies were transferred from the agar plate to 5 ml of fresh Luria-Bertani (LB) broth medium (Fisher Scientific, 244620) and grown overnight in a shaking incubator at 37 °C and 180 rpm.

The minimum inhibitory concentration (MIC) experiments were performed by diluting the overnight bacterial suspensions in sterile LB media to a final concentration of 3 × 10^4^ CFUs/ml and adding 50 μL of these diluted suspensions to individual wells of sterile 96-well plates, already containing 150 μL of different concentrations of SA in LB media and ≈3.5% of ethanol. The plates were placed in a FilterMax F5 Multimode Plate Reader (Molecular Devices) and the optical density (at 595 nm) was screened within each individual well for 24 h at 37 °C. The MIC 50 and MIC 90 values were calculated as the minimum SA concentration that prevented an optical density increase above 50 and 10%, as compared to bacteria suspensions containing only ≈3.5% ethanol in LB media. After incubation, 30 μL of the bacterial suspensions from the individual wells where no growth was observed, were transferred to sterile agar plates and incubated for 24 h. The MBC was calculated as the minimum concentration that did not allow any bacterial growth on the agar plate. We also investigated the independent effect of pH on bacterial viability by adding hydrochloric acid to LB solutions inoculated with bacteria, in order to reach the same pH values obtained during the SA MIC experiments discussed above and assessing the optical density of the suspensions at 595 nm for 24 h at 37 °C.

The viability of *S. aureus* and *E. coli* in contact with PDMS and PDMS-SA samples was probed in both planktonic and sessile states. For this purpose, bacterial cultures were grown as discussed above, and then diluted in sterile LB media to 10^4^ CFUs/mL (or 10^5^ CFUs/mL, as specified for each data set). PDMS-SA and pristine PDMS samples (with the same diameter and thickness, to be used as controls) were sterilised under UV light for 30 min and placed in sterile six-well plates. Then 4 ml of bacteria suspensions were added to each individual well and the plates were incubated for 24 h at 37 °C. At the end of the incubation period, an aliquot of bacterial suspension was taken from each well to undergo serial dilutions and plating on sterile LB agar plates that were left overnight at 37 °C to allow CFU enumeration. In parallel, the viability of sessile bacteria was assessed on the solid samples. The process included gently rinsing three times with sterile phosphate-buffered saline (PBS 1x), followed by adding 4 ml of sterile LB medium with strong pipetting for 3 min to disperse the biofilm into the suspension. An aliquot of the bacterial suspension underwent serial dilutions and plating for CFU enumeration. Viability assays were performed in three biological and three technical triplicates.

The surface coverage and viability of bacteria attached to PDMS-SA surfaces were also investigated by confocal fluorescence and scanning electron microscopy (SEM), following incubation conditions identical to the CFU enumeration experiments discussed above.

For the scanning electron microscopy (SEM) experiments the samples were gently washed three times in PBS 1x and fixed overnight at 4 °C with 4% paraformaldehyde and 2.5% glutaraldehyde in 0.1 M phosphate buffer. Sample fixation was completed in four sequential steps using 2% osmium tetroxide, 1% tannic acid, 2% osmium tetroxide and 1% uranyl acetate solutions in water within a Biowave Pro Microwave system (Pelco) set to three cycles of 100 W microwave radiation for 20 s and subsequently cooled down for 20 s. Between each processing step, the samples were thoroughly rinsed with DI-water. After the final uranyl acetate staining, the samples were rinsed with DI-water, and progressively dehydrated with different volumetric ratios of EtOH (i.e. 30%, 50%, 70%, 90 and 100%). After dehydration, the samples were critical-point dried in CO_2_ (Quorum Technologies K850) and sputter coated with 10 nm of Au/Pd (Quorum Technologies Q150T) for SEM imaging at 10 kV using a JEOL SEM 6610 system.

For confocal fluorescence imaging, samples were initially washed three times with sterile 0.85% (w/w) NaCl solution, and subsequently cut into squares <1 cm^2^ and placed in individual wells of a 24-well plate containing sterile 0.85% (w/w) NaCl solution. The staining was performed using Live/Dead BacLight bacterial viability kit (Molecular Probes, L7012) using 15 min incubation time in the dark at room temperature. After staining, the samples were imaged in 0.85% (w/w) NaCl solution using a Zeiss LSM 880 upright confocal fluorescence microscope. Collected fluorescence images were processed using Fiji software.

### Reporting summary

Further information on research design is available in the Nature Research Reporting Summary linked to this article.

## Supplementary information

Supplementary Information

Reporting Summary

## Data Availability

The data that support the findings of this study and all custom codes are available from the corresponding author upon reasonable request.

## References

[1] Whitesides GM (2006). The origins and the future of microfluidics. Nature.

[2] Raj MK, Chakraborty S (2020). PDMS microfluidics: a mini review. J. Appl. Polym. Sci..

[3] Zhang H, Chiao M (2015). Anti-fouling coatings of poly(dimethylsiloxane) devices for biological and biomedical applications. J. Med. Biol. Eng..

[4] Chen Y, Kim YS, Tillman BW, Yeo WH, Chun Y (2018). Advances in materials for recent low-profile implantable bioelectronics. Materials.

[5] Barr S, Hill EW, Bayat A (2017). Development, fabrication and evaluation of a novel biomimetic human breast tissue derived breast implant surface. Acta Biomater..

[6] Kwak MK, Jeong H-E, Suh KY (2011). Rational design and enhanced biocompatibility of a dry adhesive medical skin patch. Adv. Mater..

[7] Malcolm RK, Edwards KL, Kiser P, Romano J, Smith TJ (2010). Advances in microbicide vaginal rings. Antivir. Res..

[8] Chen IJ, Lindner E (2007). The stability of radio-frequency plasma-treated polydimethylsiloxane surfaces. Langmuir.

[9] Morra M (1990). On the aging of oxygen plasma-treated polydimethylsiloxane surfaces. J. Colloid Interface Sci..

[10] Bayston R, Grove N, Siegel J, Lawellin D, Barsham S (1989). Prevention of hydrocephalus shunt catheter colonisation in vitro by impregnation with antimicrobials. J. Neurol. Neurosurg. Psychiatry.

[11] Mai HN (2020). Antibacterial drug-release polydimethylsiloxane coating for 3D-printing dental polymer: surface alterations and antimicrobial effects. Pharmaceuticals.

[12] Shen Q (2019). Enhanced antibacterial activity of poly (dimethylsiloxane) membranes by incorporating SiO2 microspheres generated silver nanoparticles. Nanomaterials.

[13] Kim SH (2011). Flexible, stretchable and implantable PDMS encapsulated cable for implantable medical device. Biomed. Eng. Lett..

[14] Sorzabal-Bellido I (2019). Exploiting covalent, H-bonding, and π–π interactions to design antibacterial PDMS interfaces that load and release salicylic acid. ACS Appl. Bio Mater..

[15] Lee JN, Park C, Whitesides GM (2003). Solvent compatibility of poly(dimethylsiloxane)-based microfluidic devices. Anal. Chem..

[16] Humbert B, Alnot M, Quile’s F (1998). Infrared and Raman spectroscopical studies of salicylic and salicylate derivatives in aqueous solution. Spectrochim. Acta Part A.

[17] Hayat, S., Ali, B. & Ahmad, A. *Salicylic Acid: A Plant Hormone*. (eds Hayat, S. & Ahmad, A.) (Springer, 2007).

[18] Mojet BL, Ebbesen SD, Lefferts L (2010). Light at the interface: the potential of attenuated total reflection infrared spectroscopy for understanding heterogeneous catalysis in water. Chem. Soc. Rev..

[19] McVicker G (2014). Clonal expansion during Staphylococcus aureus infection dynamics reveals the effect of antibiotic intervention. PLoS Pathog..

[20] Klein EA, Gitai Z (2013). Draft genome sequence of uropathogenic Escherichia coli strain J96. Genome Announc..

[21] Flores-Mireles AL, Walker JN, Caparon M, Hultgren SJ (2015). Urinary tract infections: epidemiology, mechanisms of infection and treatment options. Nat. Rev. Microbiol..

[22] Arciola CR, Campoccia D, Montanaro L (2018). Implant infections: adhesion, biofilm formation and immune evasion. Nat. Rev. Microbiol..

[23] Susarrey-Arce A (2016). Bacterial viability on chemically modified silicon nanowire arrays. J. Mater. Chem. B.

[24] Pallavicini P (2017). Modular approach for bimodal antibacterial surfaces combining photo-switchable activity and sustained biocidal release. Sci. Rep..

