# Supplementary material for: One-step preparation of antimicrobial silicone materials based on PDMS and salicylic acid: insights from spatially and temporally resolved techniques
Source: NPJ Biofilms Microbiomes. 2021 Jun 21;7:51. doi: 10.1038/s41522-021-00223-6 (PMC8217222; doi:10.1038/s41522-021-00223-6)
Supplement: Supplementary file 1 — Supplementary Information [file 41522_2021_223_MOESM1_ESM.pdf]

Supporting Information

Title: One-step preparation of antimicrobial silicone materials based on PDMS and salicylic acid: insights from spatially and temporally resolved techniques.

Authors: Luca Barbieri, Ioritz Sorzabal Bellido, Alison J. Beckett, Ian A. Prior, Jo Fothergill, Yuri A. Diaz Fernandez, Rasmita Raval

Special Collection: Translational Research in Biofilms

Table of Content

SI1 – Additional contact angle data

SI2- Additional Raman spectroscopy data

SI3- Attenuated Total Reflection ATR-FTIR data

SI4- Effect of pH on bacterial growth

SI5- Optimisation of the model buffer

SI6- Kinetic model for the release of SA from PDMS-SA samples

SI7- Antibacterial properties of salicylic acid on the bacterial strains investigated

SI8 Antibacterial experiments at inoculum concentration 10^5 CFU/ml

SI9- Literature references for SI

SI1 – Additional contact angle data

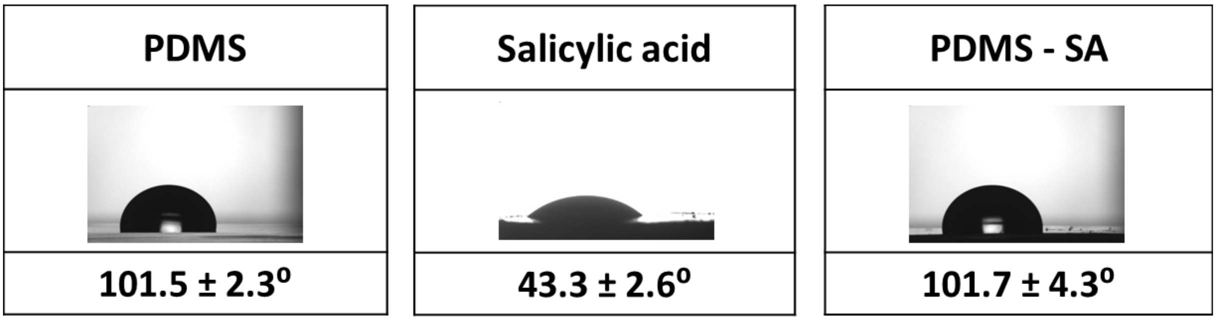

Supplementary Figure 1.1 Representative contact angle images and static contact angle values for PDMS, pure SA and PDMS-SA using citrate/citric acid model buffer. The measurements on the SA sample were performed over a pressed pellet of SA powder that provided a smooth surface.

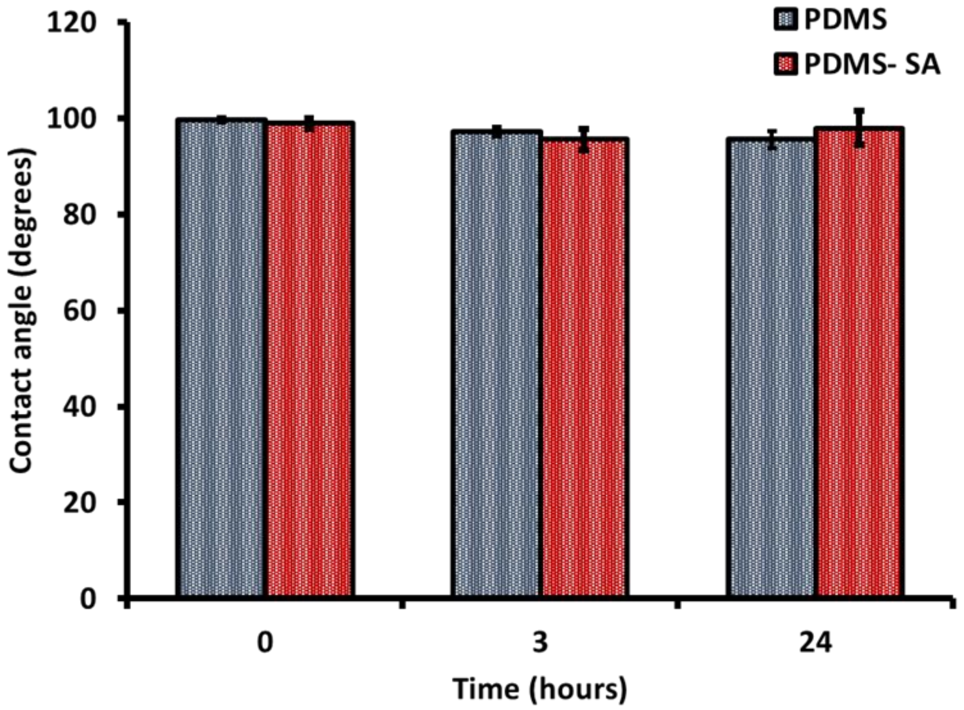

Supplementary Figure 1.2 Static contact angle values for pristine PDMS and PDMS-SA measured on dry samples (0h) and on samples exposed for 3 and 24 h to the model buffer and then quickly dried over absorbent paper and measured. The time on the horizontal axis indicates the exposure time during the pre-treatment with the model buffer, assuming that 0h has not being exposed. These time points covered the two dynamic regimes for the SA release, confirming that the permeability of PDMS-SA samples is not related to a change on the wettability of the surface.

SI2- Additional Raman spectroscopy data

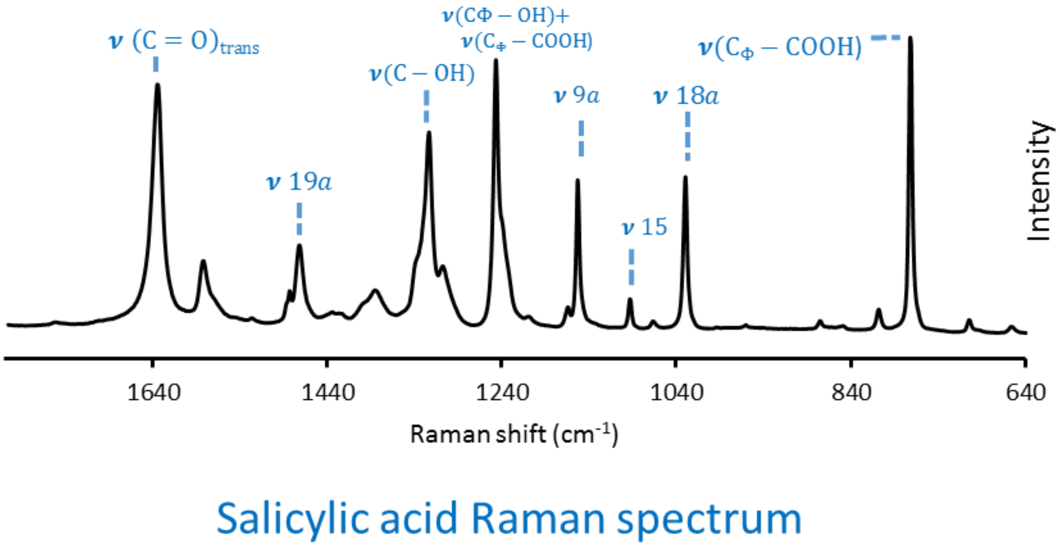

Supplementary Figure 2.1. Raman spectrum of salicylic acid, showing peak assignment according to reference [1S].

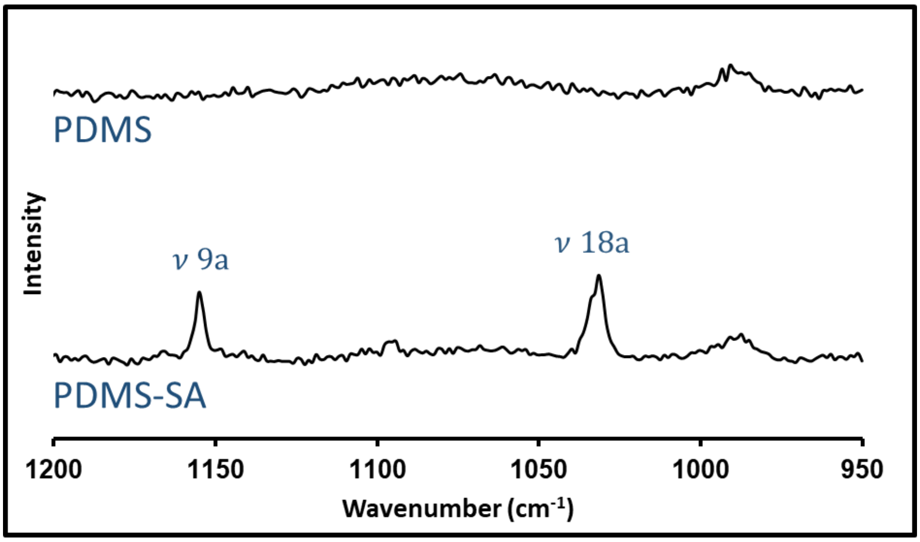

Supplementary Figure 2.2 Raman spectra of pristine PDMS and PDMS-SA, taken at the surface of the samples, showing the close up of the region between 950 and 1200 cm^{-1}, showing the residual peaks of SA at the surface of PDMS-SA, with very low intensity. The nomenclature ν 9a and ν 18a indicates that the peaks are related to the stretching of the C atoms in the aromatic ring.[1S]

SI3- Attenuated Total Reflection ATR-FTIR data

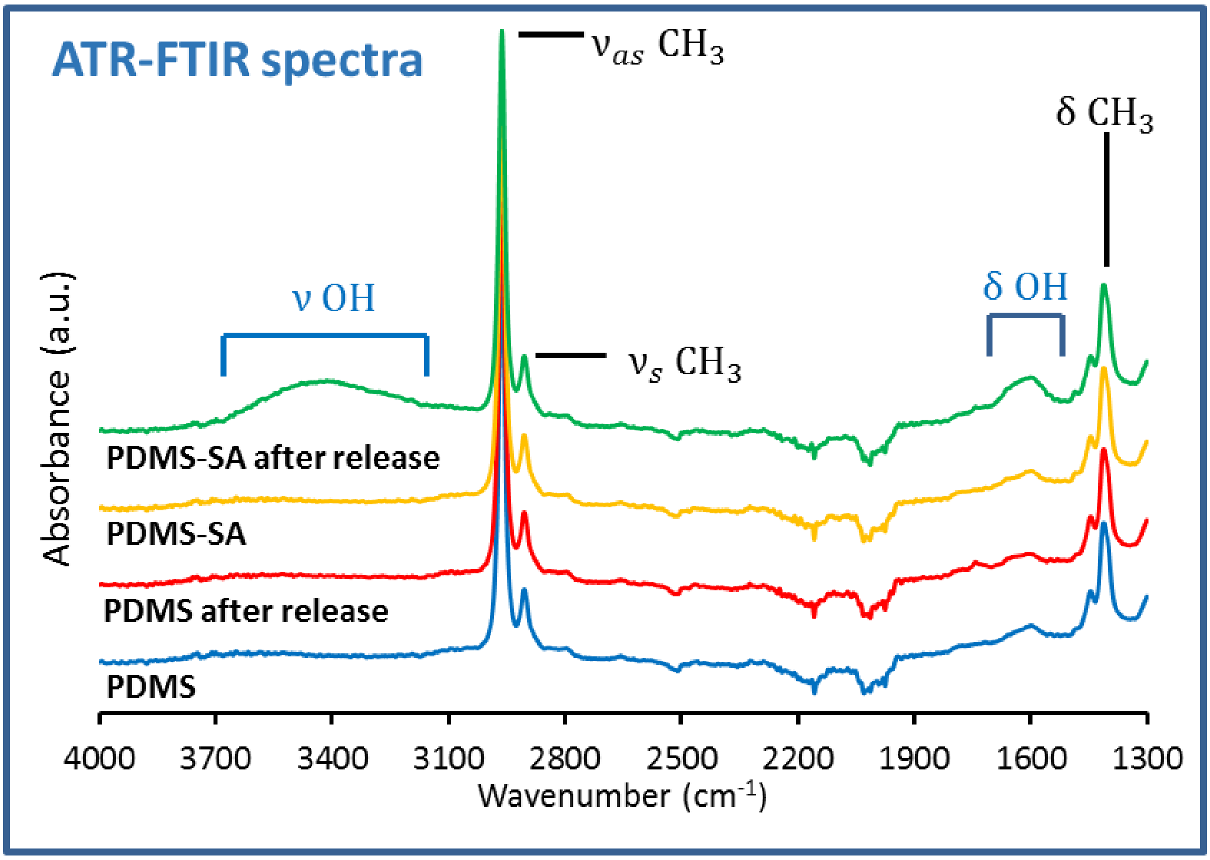

Supplementary Figure 3.1. ATR-FTIR spectra of PDMS and PDMS-SA samples before and after 24h exposure to the aqueous model buffer, showing the presence of water OH band exclusively on PDMS-SA water-exposed sample. After exposure to the liquid media, the samples were quickly dried over absorbent paper and measured immediately. This evidence demonstrates that water can permeate PDMS-SA. It is also important to note that SA peaks were not observed due to the fact that the biocide is mainly located within the sample bulk, over 100µm away from the surface and beyond the depth penetration of ATR-FTIR.

SI4- Effect of pH on bacterial growth

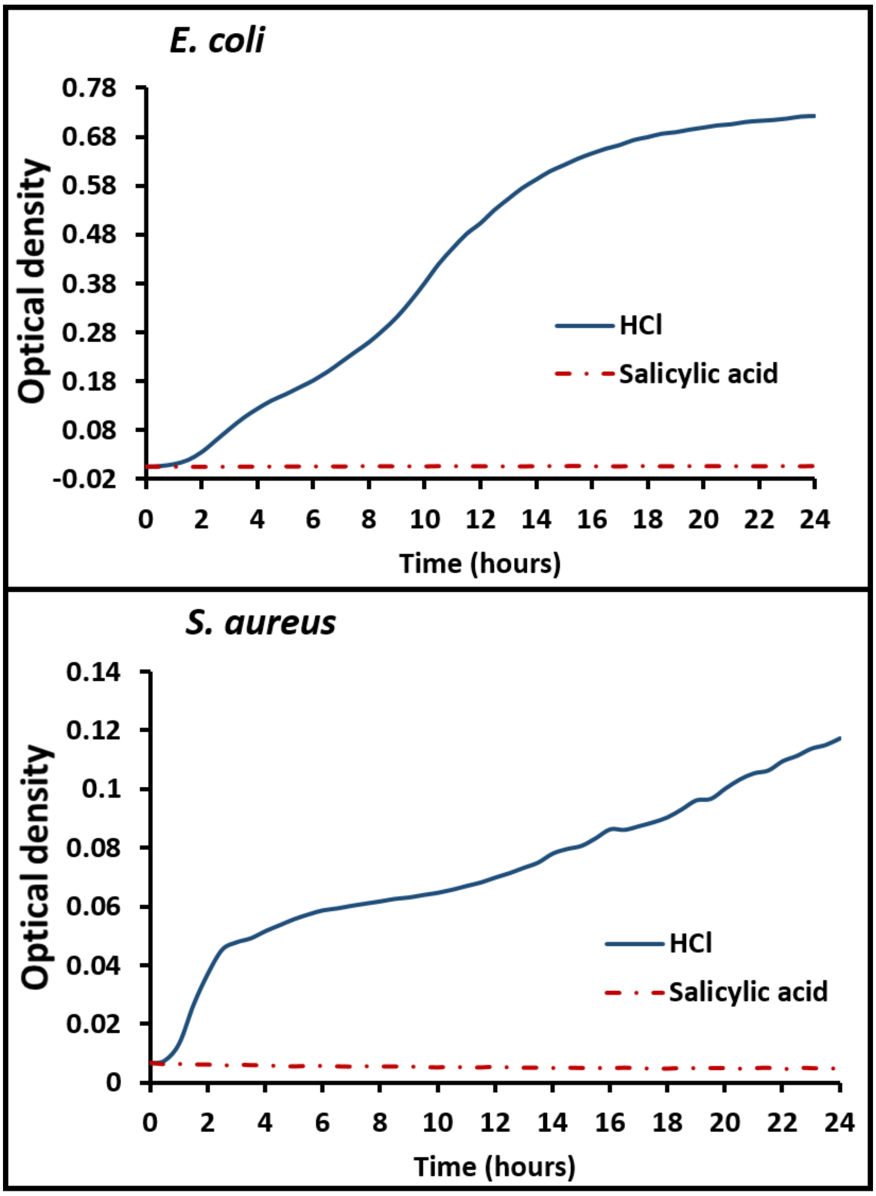

Supplementary Figure 4.1 Bacterial growth as measured by the optical density of suspensions measured over 24 hours. *E. coli* J96 (above) and *S. aureus* SH1000 (below) were grown in LB medium at the MBC of SA for each species (red) and at the same pH in the presence of HCl (blue)

SI5- Optimisation of the model buffer

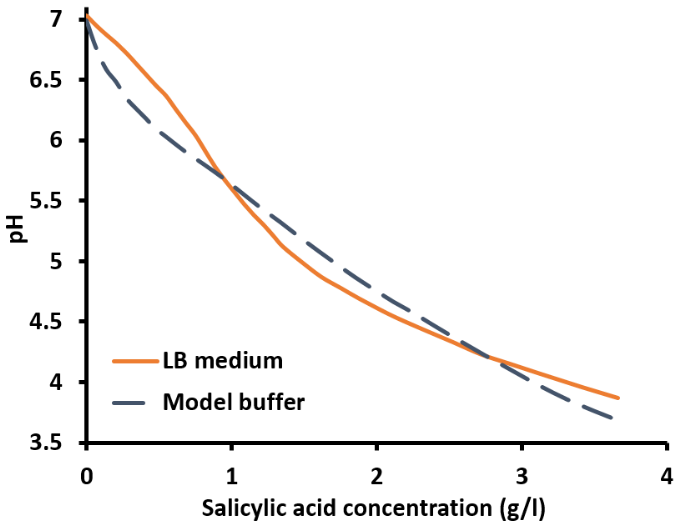

Supplementary Figure 5.1 pH profile of 11.5 mM citrate and citric acid model buffer and LB growth media at different concentrations of SA. For the range of SA concentrations investigated, the model buffer reproduces the pH response of LB media, while being optically transparent in the UV-Vis spectral region, allowing spectroscopic quantification of released SA from PDMS-SA samples.

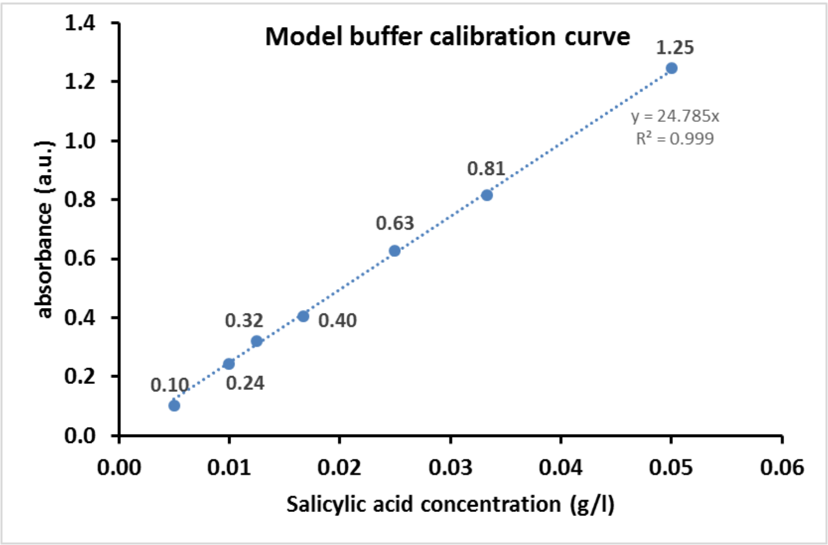

Supplementary Figure 5.2 Spectrophotometric calibration curve for SA in the model buffer used for the determination of the extinction coefficient of SA, ε=24.78 L/g at 299nm.

SI6- Kinetic model for the release of SA from PDMS-SA samples

In the main text we have shown that the release of SA from PDMS-SA materials displayed two different kinetic regimes, both characterised by the first-order time-dependent equation:

$$\frac{dC_{SA}}{dt} = k_i \cdot (S_{SA} - C_{SA}) \qquad \text{Eq.1}$$

Where C_{SA} is the time-dependent concentration of biocide in the liquid media, S_{SA} is the solubility of SA, and k_i is the apparent kinetic constant for the release process at the specific kinetic regime, i.e. k_1 for t<22h and k_2 for t>22h, both following Eq.1 in different time intervals. This expression resembles a saturation-limited solubilisation process, in which the rate of release decreases as the concentration of the solute in the media approaches the solubility and becomes effectively zero when equilibrium is reached (i.e. the equilibrium condition is $C_{SA} = S_{SA}$ at $t = \infty$).

The general analytical solution for Eq.1 leads to an exponential function with the incorporation of an integration constant (B):

$$C_{SA} = S_{SA} \cdot (1 - B \cdot e^{-k_i \cdot t}) \qquad \text{Eq.2}$$

This expression can be linearized as follows:

$$ln\left(1 - \frac{C_{SA}}{S_{SA}}\right) = -k_i \cdot t + ln(B) \qquad \text{Eq.3}$$

$$ln(\Delta C) = -k_i \cdot t + ln(B) \qquad \text{Eq.4}$$

Here the linearization variable $\Delta C = 1 - \frac{C_{SA}}{S_{SA}}$ has been introduced for simplicity.

We determined experimentally the solubility of SA in the model buffer, obtaining S_{SA}=5.43g/L and using Eq.4 we can fit the experimental data to obtain the apparent rate constants for SA release for each kinetic regime (Supplementary Figure 6.1)

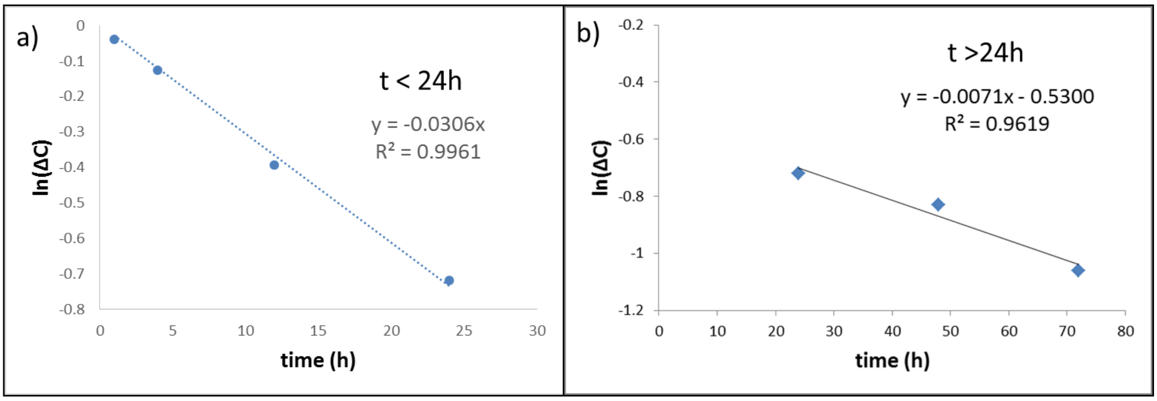

Supplementary Figure 6.1. Numerical fitting of the linearized experimental data for the release profiles for the two kinetic regimes above and below the critical time of 22h.

The high correlation observed in Supplementary Figure 6.1 for our experimental data using the kinetic model discussed above suggests that the model is able to describe the release profile of SA from PDMS-SA samples as a saturation-dependent solubilisation process. Using this model, we determined the apparent release constants, k_1=0.030s^{-1} and k_2 =0.007s^{-1} for t<22h and t>22h respectively. The two kinetic regimes can be rationalised considering the change in the pH of the solutions as function of time, when SA is released, determining the fast release of the salicylate deprotonated form at time points below 22h (pH>4) and the slower release of a mixture of salicylate and salicylic acid after 22h (pH<4). This critical time ≈22h and pH≈4 are associated with the buffering region of SA at pH≈pKa ±1, considering SA pKa=2.98.[2S]

SI7- Antibacterial properties of salicylic acid on the bacterial strains investigated

We determined the effect of salicylic acid on the two bacterial strains used in this work, namely *S. aureus* strain SH1000 and *E. coli* strain J96. These strains have been widely used as models for clinical infections.[3S-5S] For each strain, the concentrations resulting in a 50% and a 90% of growth inhibition in the planktonic state are reported in Supplementary Table 1 as the MIC50 and MIC90, respectively. In addition, we also determined the minimum bactericidal concentrations (MBC) for both strains. Details are discussed in the experimental section.

Supplementary Table 1 Minimum inhibitory concentrations (MIC50 & MIC90) and minimum bactericidal concentrations (MBC) for SA on the bacterial strains investigated.

	E. coli J96	*S. aureus* SH1000
MIC50	0.75 g/L	0.75 g/L
MIC90	1.00 g/L	1.25 g/L
MBC	2.00 g/L	1.75 g/L

SI8 Antibacterial experiments at inoculum concentrations 10^5 CFU/ml

In addition to the bacterial inhibition data presented in the main text for inoculum 10^4 CFU/ml, we also performed experiments using an inoculum of 10^5 CFU/ml. Also in this case, a significant antimicrobial effect was observed (see Supplementary Figures 8.1 & 8.2).

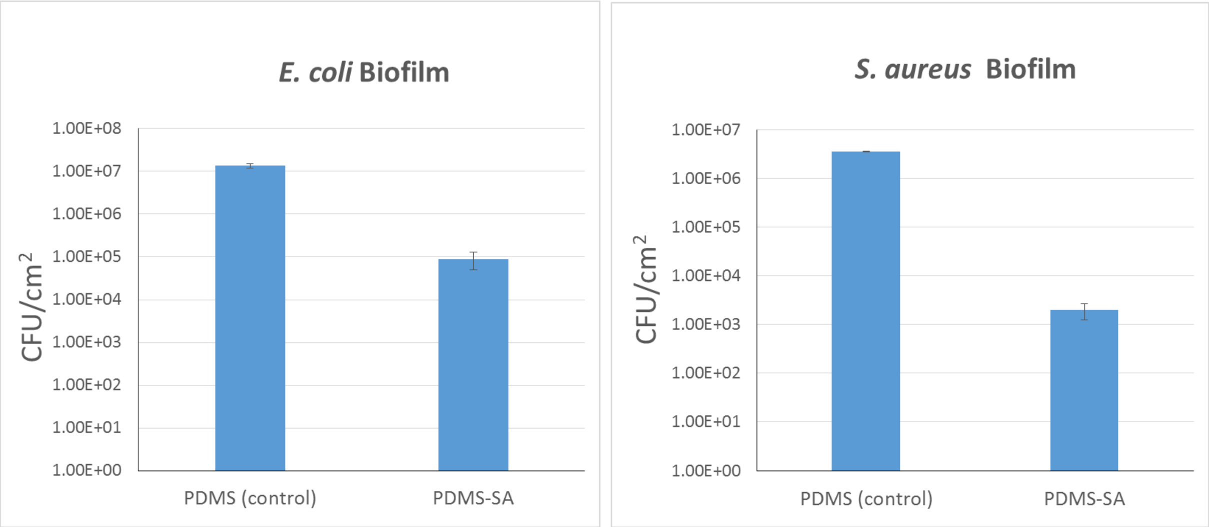

Supplementary Figure 8.1 Inhibition of sessile *E. coli* and *S. aureus* cells after 24h of incubation on PDMS-SA (inoculum 10^5 CFU/ml).

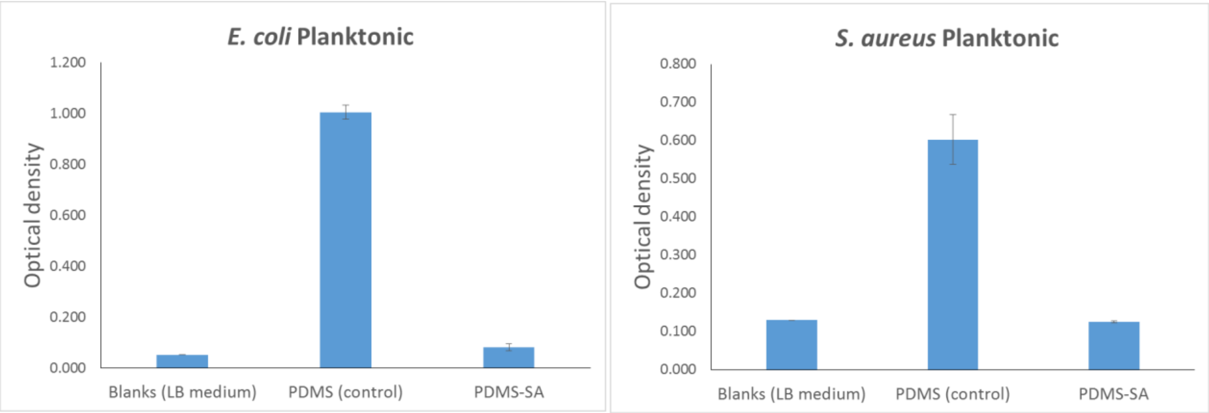

Supplementary Figure 8.2 Inhibition of planktonic *E. coli* and *S. aureus* cells after 24h of incubation on PDMS-SA (inoculum 10^5 CFU/ml).

SI9- Supplementary References

1S- Humbert, B., Alnot, M., Quile`s, F. Infrared and Raman spectroscopical studies of salicylic and salicylate derivatives in aqueous solution. *Spectrochimica Acta Part A* (1998) **54**, 465–476

2S- Hayat, S., Ali, B., Ahmad, A. Salicylic Acid: Biosynthesis, Metabolism and Physiological Role in Plants. In: Hayat S., Ahmad A. (eds) *Salicylic Acid: A Plant Hormone.* Springer (2007), Dordrecht. https://doi.org/10.1007/1-4020-5184-0_1

3S- McVicker, G., Prajsnar, T.K., Williams, A., Wagner, N.L., Boots, M., Renshaw, S.A. Clonal Expansion during Staphylococcus aureus Infection Dynamics Reveals the Effect of Antibiotic Intervention. *PLoS Pathog* (2014) **10(2)**: e1003959. https://doi.org/10.1371/journal.ppat.1003959

4S-Klein, E. A., Gitai, Z. Draft Genome Sequence of Uropathogenic Escherichia coli Strain J96. *Genome announcements* (2013) **1(1)**, e00245-12. https://doi.org/10.1128/genomeA.00245-12

5S- Flores-Mireles, A.L., Walker, J.N., Caparon, M., Hultgren, S.J. Urinary tract infections: epidemiology, mechanisms of infection and treatment options. *Nature Reviews Microbiology* (2015) **13(5)** 269–284
